# A novel phenotypic dissimilarity method for image-based high-throughput screens

**DOI:** 10.1186/1471-2105-14-336

**Published:** 2013-11-21

**Authors:** Xian Zhang, Michael Boutros

**Affiliations:** 1 German Cancer Research Center (DKFZ), Div. Signaling and Functional Genomics and Department of Cell and Molecular Biology, Medical Faculty Mannheim, Im Neuenheimer Feld 580, D-69120 Heidelberg, Germany; 2 Current address: Novartis Institutes for BioMedical Research, Basel, Switzerland

**Keywords:** Phenotypic dissimilarity, Image-based high-throughput screening, High-content screening, RNAi, Gene networks

## Abstract

**Background:**

Discovering functional relationships of genes through cell-based phenotyping has become an important approach in functional genomics. High-throughput imaging offers the ability to quantitatively assess complex phenotypes after perturbation by RNA interference (RNAi). Such image-based high-throughput RNAi screening studies have facilitated the discovery of novel components of gene networks and their interactions. Images generated by automated microscopy are typically analyzed by extracting quantitative features of individual cells, resulting in large multidimensional data sets. Robust and sensitive methods to interpret these data sets and to derive biologically relevant information in a high-throughput and unbiased manner remain to be developed.

**Results:**

Here we propose a new analysis method, PhenoDissim, which computes the phenotypic dissimilarity between cell populations via Support Vector Machine classification and cross validation. Applying this method to a kinome RNAi screening data set, we demonstrate that the proposed method shows a good replicate reproducibility, separation of controls and clustering quality, and we are able to identify siRNA phenotypes and discover potential functional links between genes.

**Conclusions:**

PhenoDissim is a novel analysis method for image-based high-throughput screen, relying on two parameters which can be automatically optimized without *a priori* knowledge. PhenoDissim is freely available as an R package.

## Background

**Figure 1:**
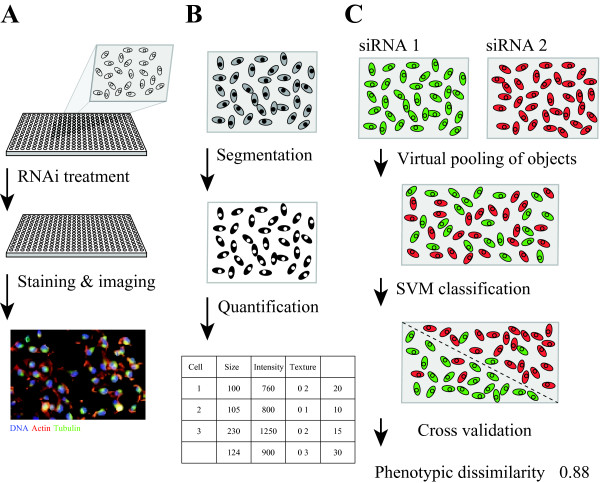
**Workflow for image-based screening, image quantification and phenotypic dissimilarity measure with SVM classification accuracy.A)** Cells are seeded into 384-well plates and treated with siRNA by reverse transfection. After incubation for 48 hours, cells are fixed, permeabilized and immunostained for DNA, tubulin and actin and imaged with an automated microscope. **B)** Cell images are processed with nucleus and cell segmentation using the R packages EBImage and imageHTS. Each cell is represented by a 46 image-based feature vector. Every treatment generates a data matrix X[m,n], where m is the number of cells and n is the number of features. **C)** For each pair of RNAi treatments, SVM classification is performed on the virtually pooled cell population based on cell features. Classification accuracy is estimated by cross validation, and defined as the phenotypic dissimilarity between treatments.

To understand phenotypes and their regulations, it is important to identify key genetic components as well as how they interact. Cell-based screening approaches have been successfully used to monitor the effect of individual gene knockdowns or small molecule treatments, identify key regulators contributing to the assessed phenotype and investigate their interactions [1,2]. Such high-throughput screening experiments can be divided into two categories: homogeneous intensity-based methods, such as reporter gene or cell viability assays, and image-based phenotyping approaches. Intensity-based methods usually report the average of cell populations, leading to scalar (or low dimensional) values per perturbation. Such screens have been designed, for example, to identify novel signaling pathway components by associating an intensity readout (e.g., luminescence or fluorescence) with a perturbation of a specific reporter gene activity [3-8]. In contrast, image-based methods mark cells with fluorescent dyes, and produce high-dimensional data sets based on images of phenotypes on a single cell level and consequently on cell populations [9-15]. Cellular phenotyping by imaging offers many advantages including flexible marker choices, subcellular resolution and ability to address cell population heterogeneity (Figure 1A), but also pose new challenges such as lower throughput, more complex infrastructure, and in particular, challenges in data analysis [16].

While the analysis of univariate readouts from intensity-based screens has been greatly facilitated by the development of specific algorithms and analysis methods [17-20], how to effectively analyze image-based phenotypes is still being explored. In general, the analysis comprises two steps: image quantification and phenotype-based analysis of gene networks. The image quantification step, which includes image pre-processing, cellular object segmentation and feature extraction, is relatively well established with several software tools offering automated, scalable and interactive pipelines [21,22]. This step generates a multidimensional data set containing cell feature information for typically 100–10000 cells per treatment and 10–200 features measured per cell (Figure 1B). The second step, to derive functional relationships from these complex datasets representing phenotypes, remains challenging. While intuitively this is performed in any kind of genetic screens, e.g. in forward genetic screens in *Drosophila melanogaster* or *Caenorhabditis elegans*, a systematic implementation for quantitative cellular image data sets is still missing. One key question is how to define quantitatives to represent the phenotype of a given perturbation based on the multidimensional cell feature data sets, before one can identify and potentially cluster phenotypes by similarity.

Previous studies typically first applied a dimension reduction or data transformation method, such as principle component analysis [23], Kolmogorov-Smirnov statistics [9], Support Vector Machine [12,24], or factor analysis [10], and generate a single feature vector for each perturbation treatment, i.e. a phenotypic profile. Then the distance between feature vectors is computed based on a distance measure, such as Euclidean distance. Although these approaches have been successfully applied in various image-based analysis, they often require manually curated training data sets and/or multiple optimization steps. Thus, for a new image-based screen campaign, selecting and optimizing the appropriate method to perform hit identification and clustering analysis remains challenging.

Here, we propose a novel method to measure phenotypic dissimilarity between cell populations in imaging screens, based on cell classification and cross validation. We define the phenotypic dissimilarity between a perturbation and a control, or between two perturbations, as the classification accuracy between the two corresponding cell populations. First, we virtually pool cells from both populations. Then, using Support Vector Machine (SVM) classification, the mixed cell population is classified into two groups based on quantitative cell features. The classification accuracy can be estimated by cross validation with the original cell labels, and defined as the phenotypic dissimilarity. A higher accuracy indicates better separation between the cell populations, thus a larger phenotypic dissimilarity. Evaluated on a kinome-wide RNAi screen for cell morphology [12], the proposed phenotypic dissimilarity method (hereafter, PhenoDissim) was able to identify RNAi perturbations causing distinct morphology phenotypes, such as siPLK1, siCOPB2 and siAKAP7. We then clustered the phenotypes based on their pair-wise dissimilarity, and genes that clustered together were functionally related.

The PhenoDissim method is relatively straightforward to apply on different high-throughput screening experiments, as it has only two parameters for SVM classification: cost and gamma, and parameter optimization can be automated. The method, as well as the quality metrics for evaluation, is implemented in a freely available R/Bioconductor package phenoDist (http://www.bioconductor.org/packages/release/bioc/html/phenoDist.html), a toolbox for data analysis in image-based high-throughput screening.

## Results

We used a previously generated image-based RNAi screening data set as a benchmark for phenotypic dissimilarity analysis [12]. The genome-wide kinase screen was conducted in duplicates using a cervix carcinoma cell line (HeLa). Cells were stained with cytoskeletal and nuclear markers (DNA, actin and tubulin) [12]. Plate layout is listed in Additional file 1: Table S4. We reanalyzed the images with the R/Bioconductor package imageHTS, and measured 46 image-based features for every cell including geometric features, Haralick texture features and Zernike moments (see Additional file 1: Table S1 for a list of all features).

### Phenotype identification with PhenoDissim

**Figure 2:**
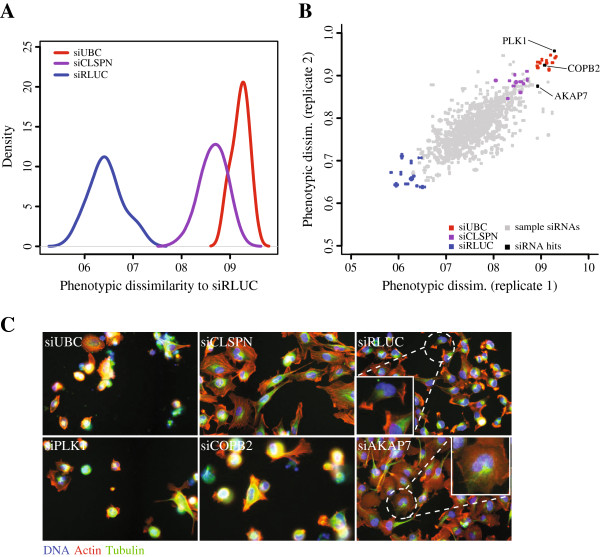
**Phenotype identification with PhenoDissim.A)** Distributions of phenotypic dissimilarity of the controls, with siUBC (red), siCLSPN (purple) and siRLUC (blue). **B)** The correlation between two replicates. Replicate 1 of all treatments including samples and controls is plotted on the X axis and replicate 2 on the Y axis. siUBC treatments are in red, siCLSPN in purple and siRLUC in blue. All samples are in gray, with the strongest phenotypes in black and labeled with gene names. **C)** Cell images of the controls (siUBC, siCLSPN, siUBC) and three phenotype hits (siPLK1, siCOPB2, siAKAP7).

One major goal in image-based screens is to identify perturbations that show significantly different phenotypes when compared to negative controls. Applying the PhenoDissim method, we computed the phenotypic dissimilarity between each perturbation and the negative controls, which indicates how significant the phenotype is (see Methods for details). The screening data set has one negative control (siRLUC) and two positive controls (siUBC and siCLSPN), with four wells of each control per 384-well plate. Figure 2A plots the distribution of the phenotypic dissimilarity of these control wells to negative control wells. Since siRLUC is the negative control, these show a low phenotypic dissimilarity (0.64±0.03). It is larger than 0.5 due to noise and cell population variation within siRLUC wells. The positive controls siUBC and siCLSPN have much higher phenotypic scores (0.92±0.02 and 0.87±0.02 respectively) and are well separated from the negative control siRLUC (Z’ factor values 0.56 and 0.40 respectively).

Phenotypic dissimilarity of all perturbations in the screen to the siRLUC control are plotted in Figure 2B with replicate 1 on the X and replicate 2 on the Y axis. The data point and error bars represent the mean and standard deviation of three independent calculations, and in most cases the error bars are negligible. There is a good correlation between biological replicates (Pearson correlation coefficient 0.75). Each control (siRLUC, siCLSPN and siUBC) is represented by 12 data points as there are three plates and four wells for each control per plate. Data points of the same control cluster together, and negative and positive controls are well separated, consistent with the density plot in Figure 2A. There are a total of 779 siRNA samples, with diverse phenotypic dissimilarity ranging from 0.65 to 0.94.

Three example perturbation with distinct phenotypes are shown in Figure 2C (siPLK1, siCOPB2 and siAKAP7). PLK1 and COPB2 are essential genes which cause viability defects similar to UBC when depleted by siRNAs. Cells treated with AKAP7 siRNAs display a morphology phenotype whereby the cell shape is more round and actin signal is more evenly distributed over the whole cytoplasm. This phenotype is consistent with previous observations that AKAP7, which encodes for A-kinase Anchoring Protein 7, localizes to cortical actin cytoskeleton under the cell membrane and when mutated, spreads to the cytoplasm [25,26].

**Figure 3:**
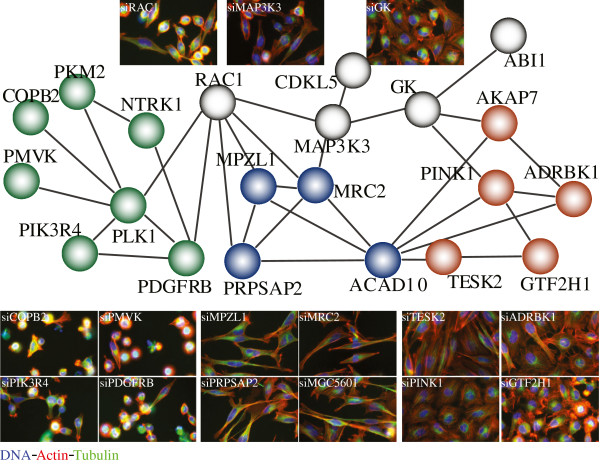
**A network of identified phenotypes and their phenotypic dissimilarity.** 31 siRNAs are identified as phenotype hits and calculated for pair-wise phenotypic dissimilarity. Each siRNA is represented by a node. siRNA pairs with phenotypic dissimilarity smaller than 0.82 are connected with an edge, with only connected nodes shown. Cell images for representative phenotypes are shown and labeled with the corresponding siRNAs.

In total, 31 siRNA perturbations (averaging two replicates of each gene) showing high phenotypic dissimilarity to siRLUC control (>0.85) indicate morphological phenotypes. With the pair-wise phenotypic dissimilarity for the 31 siRNA samples, we generated a network of phenotypes with nodes representing each phenotype and edges for phenotype dissimilarity between nodes as in Figure 3 (only phenotypic dissimilarity smaller than 0.82 and connected nodes are shown), as well as representative cell images. From network connectivity and visual inspection, we found three major groups of phenotypes. Genes highlighted in green are essential genes, and cause viability defect when knocked down. Within this group are genes PLK1 and COPB2, but also other genes such as PKM2 and PMVK. Genes highlighted in blue cause cell shape defect when depleted by siRNA. Cells are often elongated with thin stretches, suggesting defect in cell structure maintenance. Genes highlighted in orange cause strong actin staining and also affect cell shape. Genes in gray show intermediate phenotypes between the major groups. For example, siRAC1 treated cells show both a slight viability defect and an elongated shape. Further experiments to explain the underlying basis of these phenotypes are needed, however, in some cases previous functional characterizations support the observed phenotypes and their mechanism. For example, MRC2 was previously shown to be responsible for the turn-over of collagen [27] and higher levels of collagen was associated with elongated cell shapes [28]. TESK2 was shown to be involved in actin cytoskeletal organization [29]. It should also be noted that the same morphology phenotype can be caused by unrelated mechanisms, nevertheless, grouping similar phenotypes may help identify and understand functionally related genes and their interactions.

### Gene clustering analysis with PhenoDissim

**Figure 4:**
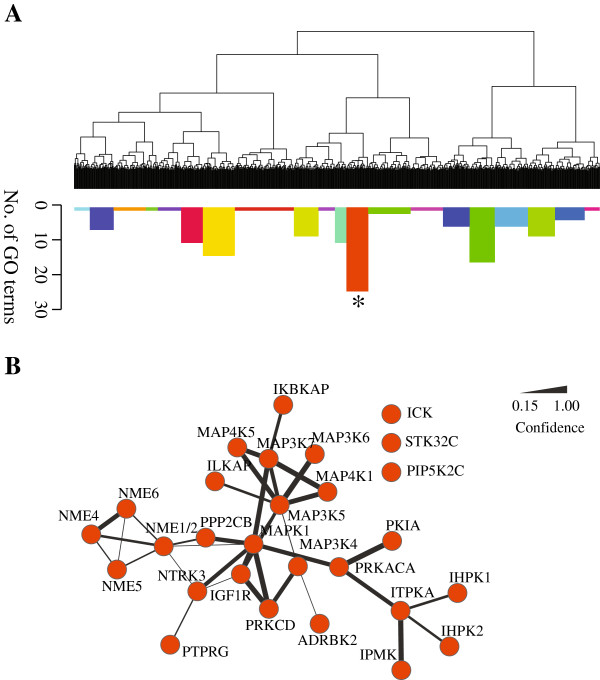
**Phenotypic clustering with PhenoDissim.A)** The phenotypic clustering tree is plotted as dendrogram. The hierarchical tree is cut into 20 clusters, with each cluster analyzed for the number of enriched GO terms (indicated by the color and height of the bars). The cluster with the most enriched GO terms is marked by an asterisk. **B)** Genes from the marked cluster are shown with gene interactions retrieved from the STRING interaction database, where nodes represent genes in the cluster and edges represent interactions identified by the STRING database (edges are weighted based on the evidence score).

We then clustered genes by pair-wise phenotypic dissimilarity of the whole screening set to identify genes that perform potentially related functions. To this end, we averaged the two replicates and generated a 779×779 phenotypic dissimilarity matrix, with each row and each column representing an siRNA treatment. Then hierarchical clustering was performed based on the phenotypic dissimilarity matrix (shown as a dendrogram in Figure 4A). The clustering tree was cut into 20 clusters, and each cluster analyzed for GO term enrichment (see Methods for details). The clusters are shown as colored bars, and the height of each bar indicates how many enriched GO terms found in the corresponding cluster (Figure 4A). There are a total of 126 enriched GO terms identified, with clusters vary in the number of enriched GO terms.

Genes from the cluster with the highest number of enriched GO terms (marked with an asterisk in Figure 4A) are shown in Figure 4B, where nodes represent gene members and edges represent the gene-gene interaction identified in the STRING database [29]. The weight of each edge is proportional to interaction confidence. 29 of 32 genes were found to be connected in STRING. Particularly, we noticed two functional groups, the mitogen-activated protein kinase (MAPK) signaling pathway and the protein expressed in non-metastatic cells (NME) family. The MAPK pathway is involved in multiple cellular functions including proliferation, differentiation and migration [30]. Eight members of the MAPK pathway are found in this cluster, MAP3K4, MAP3K5, MAP3K6, MAP3K7, MAP4K1, MAP4K5, MAPK1, PRKACA. These genes show phenotypic similarity among each other, and the associated GO terms are enriched such as activation of JUN kinase activity (GO:0007257, p value 0.002, odds ratio 10). The NME gene family was discovered as a metastasis suppressor [31], and was later shown to be involved in cell proliferation and differentiation [32,33] as well. Five NME genes are present in this cluster and the associated GO terms are enriched such as GTP biosynthetic process (GO:0006183, p value 1×10^−5^, odds ratio 33). Grouping genes from the same pathway or family together validates the clustering analysis. Additionally, the MAPK pathway and the NME gene family are clustered together, which may suggest a functional link. This is supported by the previous finding that overexpression of NME represses MAPK phosphorylation, and thus inhibits cell migration and metastasis [34,35]. In summary, our analysis has shown that clustering based on the PhenoDissim method has the potential to identify gene functional clusters.

## Discussion

The generation of phenotype-based perturbation networks based on cellular phenotyping is becoming a powerful approach in systems biology, functional genomics and drug discovery [36]. Several approaches have been developed to quantify image-based readouts from image-based screening via segmentation and feature extraction [22,37,38]. However, translating multidimensional cell feature data into phenotypic information remains elusive and hinders the further application of image-based screening. Previous studies have proposed multiple analysis methods with different dimension reduction and statistical learning algorithms [9,10,12,24], but these methods often rely on human experts to provide biological knowledge, such as for feature selection and training data set annotation. These approaches also require the optimization of multiple parameters, which prevents an easy adaptation to other image-based screens with different setups.

We have developed here a new phenotypic dissimilarity measure, PhenoDissim, for image-based high-throughput screening. The proposed method can identify phenotypes by computing the dissimilarity between samples and controls, and determine phenotype-based gene networks by computing the dissimilarity between samples. With the proposed method, we have identified distinct phenotypes, and functionally related genes by cluster analysis. This method only requires the optimization of SVM classification parameters cost and gamma, without knowing cell lines, fluorescent markers, treatment types or biological questions.

Comparing the performance of PhenoDissim with previous methods is challenging due to the lack of gold standards and different scales of screening data sets. We have designed quality metrics to assess replicate reproducibility and separation of controls, which provide evaluation of the whole screen from different perspectives (see Methods for details). As summarized in Table 1, the PhenoDissim method performs similarly or better on the benchmark data set compared with previous methods, in terms of replicate reproducibility, separation of controls and gene clustering quality. It should be noted that we have not extensively performed optimization for other methods, indicating that they could show a higher performance when fine tuned. In addition other data sets may behave differently for other analysis methods.

**Table 1:** Evaluation of analysis methods

**Method**	**Replicate**	** Z’ factor**		**GO enrichment**
	**correlation**	**siUBC**	**siCLSPN**	
PCA	0.74	-0.74	-1.31	88
Factor analysis	0.39	-1.87	-0.12	92
KS statistic	0.66	0.07	0.21	51
SVM weight vector	0.07	-84.94	-3.49	101
SVM supervised	0.75	-0.29	-1.34	83
PhenoDissim*	0.75±0.001	0.56±0.01	0.40±0.01	131±18

* mean ± standard deviation for three independent runs.

Because PhenoDissim applies classification accuracy as a proxy for dissimilarity, the highest dissimilarity value is 1, or 100% classification accuracy. Thus PhenoDissim will not be able to quantify the differences between two phenotypes if both have 100% classification accuracy from the negative control. Also when treatments generate similar phenotypes but different cellular subpopulation distribution, PhenoDissim might not be able to detect the distinctions. In these scenarios it will need to be combined with other methods. Because SVM classification needs to be performed between each pair of treatments, the proposed method is computationally more intensive than previous methods (O(n^2^)). We have estimated that with the data set used in this study, every dissimilarity calculation takes about 10 seconds on a 2 GHz Intel Xeon CPU (data not shown). The computation needed for each comparison will be affected by the number of features, the number of cells and the SVM parameters when applied to other data sets.

Diverse machine learning methods have been proposed for the analysis of image-based screens, which can be classified into generative model approaches, e.g. principle component analysis and factor analysis, versus discriminative model approaches, e.g. support vector machines; or supervised versus unsupervised approaches. The proposed PhenoDissim method is discriminative and unsupervised. Depending on the biological question of the screening campaign, certain type of methods may be better fit than others. Without *a priori* knowledge, PhenoDissim captures any phenotypes different from the negative controls as well as the dissimilarity between phenotypes. However, this method does not elucidate what are the phenotypes and what image-based features define the phenotypes.

## Conclusions

The proposed PhenoDissim method needs minimum parameter optimization and is successfully applied in phenotype identification and clustering in the current kinome RNAi screen. More and diverse image-based screening data sets need to be investigated to evaluate proposed analysis methods. To facilitate screen data analysis in general, we have developed an R package, available through the Bioconductor project [39] (http://www.bioconductor.org/packages/release/bioc/html/phenoDist.html), which implements analysis methods and quality metrics used in this study. As a toolbox for phenotypic analysis in image-based screening, and quality control of screens and analysis methods, the phenoDist package facilitates testing different analysis methods with various image-based screens, which will help develop accurate and effective data analysis methods, and promote further application of image-based screening.

## Methods

### Phenotypic dissimilarity measure with Support Vector Machine classification accuracy

Cell classification is to learn a mapping *X*→*Y*, where *x*∈*X* is a set of cell feature vectors and *y*∈*Y* is a cell label. Given two treatments (e.g., treated by siRNAs *i* and *j*), we collect (*x*_*i*_, *y*_*i*_) and (*x*_*j*_, *y*_*j*_), where *x*_*i*_ is a set of feature vectors representing cells from treatment *i*, *x*_*j*_ is a set of feature vectors representing cells from treatment *j*, and *y*_*i*_ can be assigned 1 and *y*_*j*_ can be assigned -1 to represent cell labeling. Virtually pooling (*x*_*i*_, *y*_*i*_) and (*x*_*j*_, *y*_*j*_), we can find a classifier *y*=*f*(*x*,*α*), where *α* is the parameter space of the function. The accuracy of the classification represents the separability of these two cell populations, and thus the phenotypic distance between the two treatments. One can estimate the classification accuracy by performing cross validation defined as *CV*($\hat{f}$, *α*)=$\frac{1}{N}\sum_{i=1}^{N}L(y_{i},{\hat{f}}^{-K(i)}(x_{i},\alpha )$, where *L* is the zero-one loss function, L(y,ŷ)=1ify≠ŷ, and 0 otherwise; *κ*{1,…,*N*}→{1,…,*K*} is an indexing function for K-fold cross validation. A support vector machine classifier performs classification in an enlarged feature space as $f(x)=\sum_{i=1}^{N}\alpha_{i}y_{i}\langle h(x),h(x_{i})\rangle +\beta_{0}$, where *h*(*x*) is the function to map the original features to an enlarged space and 〈,〉 is the dot product operator. We can define the kernel function as *K*(*x*,*x*^′^)=〈*h*(*x*),*h*(*x*^′^)〉. Two kernel functions are most frequently used, linear and radial *K*(*x*,*x*^′^)=*exp*(−*γ*∥*x*−*x*^′^∥^2^). We evaluated both kernel functions together with other methods (see below), and found that the radial kernel function always performed better than the linear kernel function (data not shown). Thus only the radial kernel function data is presented here. We first performed parameter tuning for cost (*C*) and gamma (*γ*) (see Additional file 1: Table S3). Then for every pair of treatments, an SVM classification was performed on the cells pooled from both populations (Figure 1C). The classification accuracy was estimated from five-fold cross validation and defined as the phenotypic dissimilarity, which ranged from 0.5 to 1.0, with 0.5 indicating random classification (identical phenotypes) and 1.0 indicating perfect classification (completely distinct phenotypes). To assess variation due to random sampling in cross validation, each classification and cross validation was performed three times, with average and standard deviation of three trials being reported.

### Quality metrics for evaluation

In order to quantitatively evaluate high-throughput screening experiments, we assessed replicate reproducibility, separation of controls, and gene clustering quality. We applied PhenoDissim and previous methods to the same screening data set, and the quality measurements indicated the performance of different data analysis methods.

Replicate reproducibility: In the data set, there were four negative control wells (siRLUC) per plate. For each sample well containing a perturbation, we computed the phenotypic dissimilarity between the sample well and each of the four negative controls on the same plate. There were a total of 779 genes targeted in the human kinome library with two replicates for each gene. To measure reproducibility, we calculated the Pearson correlation coefficient between replicate samples, of the sample phenotypic dissimilarity to each negative control, between replicate samples.

Separation of controls: There were two types of positive controls (siUBC and siCLSPN) and one negative control (siRLUC) in the screening data set, with each control represented by four wells per plate. For positive controls, phenotypic dissimilarity to negative control was calculated the same way as the samples. For negative controls, we computed the phenotypic dissimilarity between each negative control well and the other three negative control wells on the same plate, and averaged the three measurements. The performance of the phenotypic dissimilarity method can be indicated by the separation between negative and positive controls, which can be measured by the robust Z’ factor score, as $Z^{\prime }=1-3({\text{MAD}}_{\text{pos}}+{\text{MAD}}_{\text{neg}}/\text{abs}(\mu_{\frac{1}{2}\text{pos}}-\mu_{\frac{1}{2}\text{neg}}))$, where *MAD* is the median absolute deviation, *μ* is the median and *abs* is the absolute value.

Gene clustering quality: After averaging two replicates of the same gene, we performed hierarchical clustering of 779 genes, based on their pair-wise phenotypic dissimilarity matrix. For validation, the hierarchical tree was cut into 20 clusters (the number of clusters is determined to maximize enriched GO terms), with genes in each cluster analyzed for gene annotation enrichment. Since most genes were kinases, we used the biological process gene ontology annotation [40]. For each cluster, genes within the cluster were defined as the genes of interest and all genes in the library defined as the gene universe. Fisher’s exact test was performed and GO terms with p value smaller than 0.01 were identified as enriched [41]. The total number of enriched GO terms was used to evaluate the quality of the clustering.

### Software implementation

We implemented the presented phenotypic dissimilarity method and quality control metrics in an R/Bioconductor package, named phenoDist (http://bioconductor.org/). The presented analysis was performed with R version 2.13 and phenoDist version 1.0.0.

## Competing interests

The authors declare that they have no competing interests.

## Authors’ contributions

XZ and MB conceived the idea. XZ carried out the analysis. XZ and MB wrote the manuscript. Both authors read and approved the final manuscript.

## Supplementary Material

Additional file 1 Supplementary methods. Click here for file
