# Supplementary material for: A novel phenotypic dissimilarity method for image-based high-throughput screens
Source: BMC Bioinformatics. 2013 Nov 21;14:336. doi: 10.1186/1471-2105-14-336 (PMC4225524; doi:10.1186/1471-2105-14-336)
Supplement: Additional file 1 — Supplementary methods. [file 1471-2105-14-336-S1.pdf]

Supplementary methods

Image-based features
A total of 23 image-based features including geometric features, Haralick texture features, Zernike moments and correlations between channels are computed for the cellular mask and the nuclear mask. Thus each cell is represented by 46 features. Table S1 lists the names of these features; detailed description and formulation of the features can be found in the documentation of the R package imageHTS.

After feature extraction, we generate cell feature matrix X, with the dimensions of $k \times m \times n$, where k is the number of wells in the screen, m is the number of cells in every well and n is the number of features measured for each cell. This cell feature matrix is then analyzed with certain phenotypic dissimilarity measure.

Phenotypic dissimilarity measure
A total of five phenotypic dissimilarity measures are evaluated in this study. The PhenoDissim method is described in the main text. In the other five methods, the feature matrix X with the dimensions of $k \times m \times n$ is always transformed with different dimension reduction methods into a matrix Y, before computed for the dissimilarity matrix D with the dimensions of $k \times k$, via the Mahalanobis distance, defined as $d(p,q) = \sqrt{(p-q)^T S^{-1}(p-q)}$. Details of the dimension reduction methods are described below.

Principle component analysis
Wells are first summarized by the median of all cells, which results in the well feature matrix X, with the dimensions of $k \times n$. X is then transformed by principle component analysis as $Y = A(X - \mu_x)$. We have found that transformation with the first 30 PCA dimensions (cumulative proportion of variation: 0.996) generates the best performance (data not shown).

Factor analysis
Features of cells from different wells are first combined together into X, with the dimensions of $K \times n$, where K is the number of all cells in the screen, i.e., the product of k and m. In factor analysis, X is considered as a linear function of common factors F and unique factors ε, i.e., $X = LF + \varepsilon$, where L is the factor loadings. Different numbers of factors ranging 5 to 20 are varied to evaluate their performances (Figure S2). 12 factors are selected according to the Kaiser criterion (eigenvalues >1). Factor scores are then averaged per well and X is transformed into the factor matrix Y with the dimensions of $k \times n$.

Kolmogorov-Smirnov
Cells in each well are compared to cells from the negative control wells. For every feature, a KS test is performed between sample cells and the negative control cells, and the KS statistic (D) is reported. Thus, the feature matrix X is transformed into the KS statistic matrix Y with the dimensions of $k \times n$.

SVM weight vector

Cells in each well are compared to cells from the negative control wells by SVM classification with a linear kernel, as $f(x) = \sum_{i=1}^{N} \alpha_i y_i \langle h(x), h(x_i) \rangle + \beta_0$, where $h(x)$ is the function to map the original features to an enlarged space and $\langle , \rangle$ is the dot product operator. The linear kernel is defined as $K(x, x') = \langle x, x' \rangle$. Thus the feature matrix X is transformed into the SVM weight vector matrix Y, with the dimensions of $k \times n$.

SVM supervised classification
With manually annotated training sets, cells are classified into six classes via SVM based on the features. Combination of fractions of different cell classes per well and the median cell features to results in the matrix X' with the dimensions of $k \times (n + 6)$, which is subsequently transformed by PCA (30 dimensions) into the matrix Y with the dimensions of $k \times 30$.

SVM classification parameter tuning
SVM classification with a linear kernel is tuned for the parameter cost (C), and SVM classification with a radial kernel is tuned for parameters cost (C) and gamma (γ). The range for C is (2^{-5}, 2^{0}, 2^{5}, 2^{10}) and the range for γ is (2^{0}, 2^{-5}, 2^{-10}, 2^{-15}). 50 wells are randomly selected from the screen, and for each pair of wells we perform an SVM classification and estimated classification accuracy by five-fold cross validation. The median classification accuracy under each parameter set is reported for the linear kernel (Table S2) and the radial kernel (Table S3). The whole process is performed twice (1st set, 2nd set) to minimize random sampling effects. Balancing a high accuracy and a feasible computational cost, we select $C = 2^{0}$ and $\gamma = 2^{-5}$ as the parameter values for the following analysis.

Plate batch effect assessment
In order to assess whether plate batch effect is biasing our analysis, we plot dissimilarity values between wells from the same plate, and between wells from different plates (Figure S1). The two density plots are almost completely overlapping, with dissimilarity of wells on the same plate having a slight shift to the left. Kolmogorov-Smirnov test results D = 0.0382, p-value < 2.2e-16 shows that the two distributions are highly similar. Although p-value is significant, this is due to the large sample size (~1 million). The gap is small and should not affect our analysis results.

Supplementary Figures

Figure S1. Density plot of dissimilarity values of wells on the same plate and of wells on different plates.

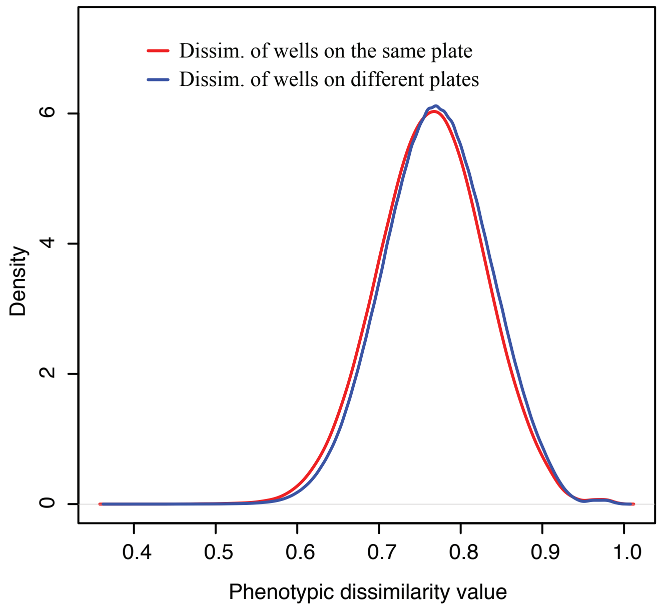

Figure S2. Performance of factor analysis when varying the number of factors.

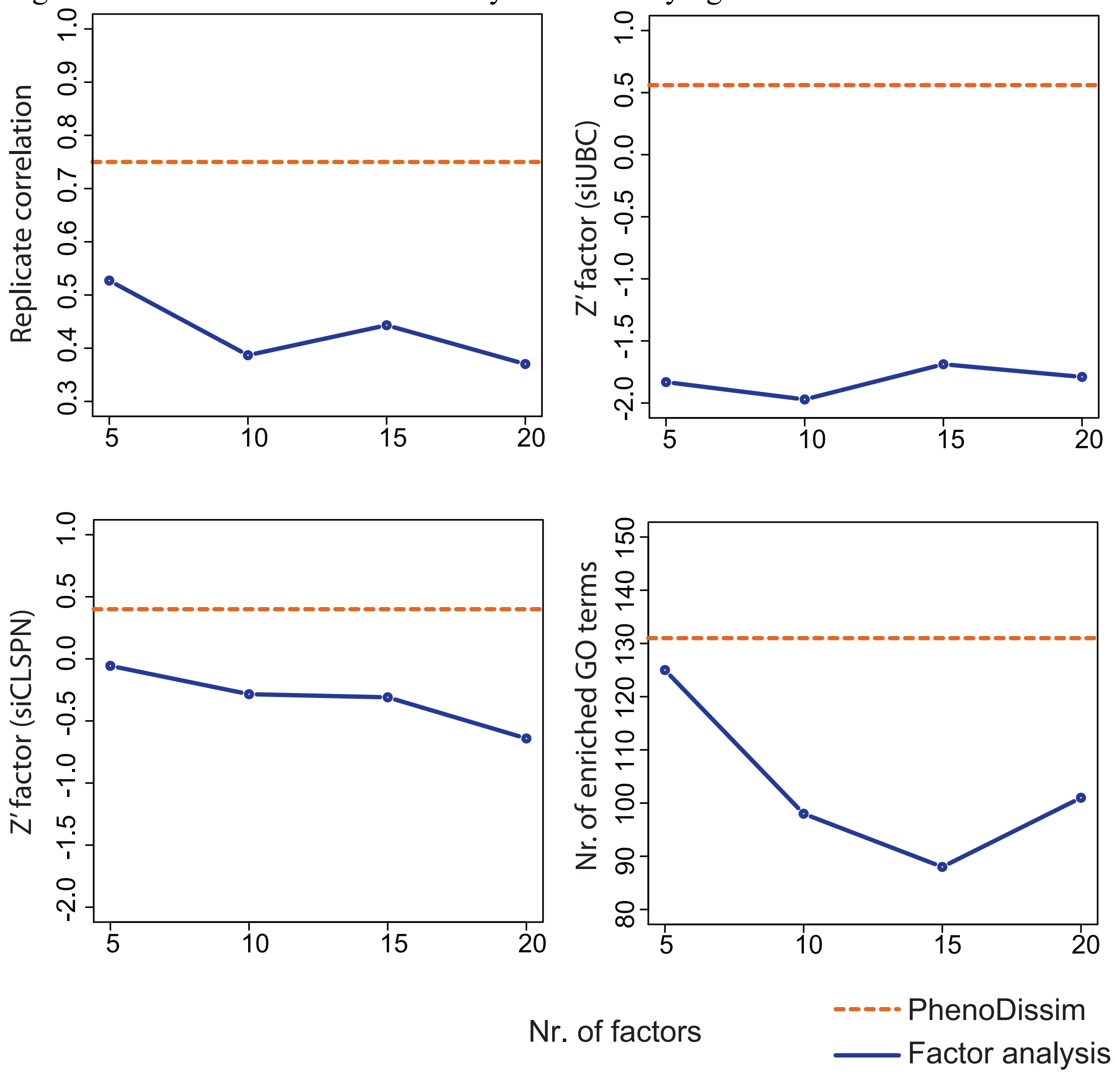

Supplementary tables

Table S1. List of image-based features.

Category	Features
Geometric features	size
	eccentricity
	actin intensity
	tubulin intensity
	hoechst intensity
Haralick texture features	actin texture
	tubulin texture
	hoechst texture
	actin contrast
	tubulin contrast
	hoechst contrast
	actin entropy
	tubulin entropy
	hoechst entropy
Zernike moments	actin angular second moment
	tubuline angular second moment
	hoechst angular second moment
	actin measure of correlation
	tubulin measure of correlation
	hoechst measure of correlation
Correlation features	actin and tubulin correlation
	actin and hoechst correlation
	tubulin and hoechst correlation

Table S2. Parameter tuning for linear kernel SVM.

	cost (C)			
	2^{-5}	2^{0}	2^{5}	2^{10}
1st set	0.74	0.77	0.78	0.78
2nd set	0.78	0.79	0.79	0.79

Table S3. Parameter tuning for radial kernel SVM.

		cost (C)			
	gamma (γ)	2^{-5}	2^{0}	2^{5}	2^{10}
1st set	2^{-15}	0.53	0.53	0.53	0.53
	2^{-10}	0.53	0.76	0.79	0.80
	2^{-5}	0.71	0.78	0.77	0.76
	2^{0}	0.53	0.53	0.53	0.53
2nd set	2^{-15}	0.54	0.54	0.73	0.76
	2^{-10}	0.54	0.73	0.78	0.80
	2^{-5}	0.67	0.77	0.77	0.76
	2^{0}	0.54	0.54	0.54	0.54

Table S4. Plate layout.

Plate	Well	Content	siRNAID	GeneID

1	A01	empty	empty	empty
1	A02	empty	empty	empty
1	A03	control		UBC
1	A04	control		PLK1
1	A05	sample	M-005300-00	AAK1
1	A06	sample	M-003104-02	AXL
1	A07	sample	M-005301-01	AATK
1	A08	sample	M-008914-00	AZU1
1	A09	sample	M-004061-00	CERK
1	A10	sample	M-004932-00	BCKDK
1	A11	sample	M-009511-00	RAPGEF4
1	A12	sample	M-003875-04	BCR
1	A13	sample	M-003255-02	CHEK1
1	A14	sample	M-005436-00	BDKRB2
1	A15	sample	M-003256-05	CHEK2
1	A16	sample	M-003105-03	BLK
1	A17	sample	M-006704-00	CHKA
1	A18	sample	M-020353-01	BLNK
1	A19	sample	M-006705-00	CHKB
1	A20	sample	M-005071-00	BMP2K
1	A21	sample	M-005462-01	CHRM1
1	A22	sample	M-004933-03	BMPR1A
1	A23	sample	M-003473-01	CHUK
1	A24	sample	M-004934-01	BMPR1B
1	B01	empty	empty	empty
1	B02	empty	empty	empty
1	B03	control		UBC
1	B04	control		PLK1
1	B05	sample	M-003257-02	PLK3
1	B06	sample	M-003126-01	ERBB2
1	B07	sample	M-012217-00	CNKSR1
1	B08	sample	M-003127-02	ERBB3
1	B09	sample	M-012101-00	COL4A3BP
1	B10	sample	M-003128-02	ERBB4
1	B11	sample	M-019847-01	COPB2
1	B12	sample	M-004807-01	ERK8
1	B13	sample	M-004031-02	CRK7
1	B14	sample	M-004951-01	ERN1
1	B15	sample	M-012023-01	CRKL
1	B16	sample	M-006530-01	EVI1
1	B17	sample	M-003109-02	CSF1R
1	B18	sample	M-005317-02	FASTK
1	B19	sample	M-003110-02	CSK
1	B20	sample	M-003129-01	FER
1	B21	sample	M-003957-03	CSNK1A1

1	B22	sample	M-003130-01	FES
1	B23	sample	M-003478-00	CSNK1D
1	B24	sample	M-003131-02	FGFR1
1	C01	empty	empty	empty
1	C02	empty	empty	empty
1	C03	control		RLUC
1	C04	control		KIF23
1	C05	sample	M-013591-01	CINP
1	C06	sample	M-005309-02	BMPR2
1	C07	sample	M-004613-00	CIT
1	C08	sample	M-003106-02	BMX
1	C09	sample	M-006706-01	CKB
1	C10	sample	M-003460-01	BRAF
1	C11	sample	M-006707-00	CKM
1	C12	sample	M-004935-01	BRD2
1	C13	sample	M-006708-00	CKMT1
1	C14	sample	M-004938-01	BRDT
1	C15	sample	M-006709-00	CKMT2
1	C16	sample	M-003107-01	BTK
1	C17	sample	M-004586-01	CKS1B
1	C18	sample	M-004102-00	BUB1
1	C19	sample	M-007678-00	CKS2
1	C20	sample	M-004101-00	BUB1B
1	C21	sample	M-004800-00	CLK1
1	C22	sample	M-005310-00	C14ORF20
1	C23	sample	M-004801-01	CLK2
1	C24	sample	M-003108-01	TP53RK
1	D01	empty	empty	empty
1	D02	empty	empty	empty
1	D03	control		RLUC
1	D04	control		KIF23
1	D05	sample	M-003479-01	CSNK1E
1	D06	sample	M-003132-01	FGFR2
1	D07	sample	M-004666-01	CSNK1G1
1	D08	sample	M-003133-01	FGFR3
1	D09	sample	M-004678-00	CSNK1G2
1	D10	sample	M-003134-01	FGFR4
1	D11	sample	M-004679-02	CSNK1G3
1	D12	sample	M-003135-02	FGR
1	D13	sample	M-003475-00	CSNK2A1
1	D14	sample	M-005318-00	FLJ10074
1	D15	sample	M-004752-00	CSNK2A2
1	D16	sample	M-005078-00	FLJ10761
1	D17	sample	M-007679-00	CSNK2B
1	D18	sample	M-007256-00	FLJ10842

1	D19	sample	M-007871-00	CXCL10
1	D20	sample	M-007260-00	RFK
1	D21	sample	M-004417-02	DAPK1
1	D22	sample	M-007726-00	FLJ12476
1	D23	sample	M-004418-02	DAPK2
1	D24	sample	M-006318-00	FLJ13052
1	E01	empty	empty	empty
1	E02	empty	empty	empty
1	E03	control		CASP1
1	E04	control		KIF11
1	E05	sample	M-004802-01	CLK3
1	E06	sample	M-003754-01	TRIB3
1	E07	sample	M-004803-00	CLK4
1	E08	sample	M-007254-01	C6ORF199
1	E09	sample	M-003100-01	ABL1
1	E10	sample	M-018324-01	C7ORF16
1	E11	sample	M-003101-01	ABL2
1	E12	sample	M-003633-01	TRIB1
1	E13	sample	M-003102-02	ACK1
1	E14	sample	M-006703-02	C9ORF12
1	E15	sample	M-004924-01	ACVR1
1	E16	sample	M-019939-01	CALM3
1	E17	sample	M-004925-01	ACVR1B
1	E18	sample	M-004940-00	CAMK1
1	E19	sample	M-004926-01	ACVR2
1	E20	sample	M-004946-00	CAMK1D
1	E21	sample	M-004927-00	ACVR2B
1	E22	sample	M-004941-01	CAMK1G
1	E23	sample	M-005302-02	ACVRL1
1	E24	sample	M-004942-00	CAMK2A
1	F01	empty	empty	empty
1	F02	empty	empty	empty
1	F03	control		CASP1
1	F04	control		KIF11
1	F05	sample	M-004947-00	DAPK3
1	F06	sample	M-004020-03	MASTL
1	F07	sample	M-004884-01	DCAMKL1
1	F08	sample	M-005319-01	FLJ20574
1	F09	sample	M-006710-00	DCK
1	F10	sample	M-007253-00	THNSL1
1	F11	sample	M-003111-03	DDR1
1	F12	sample	M-004843-00	FLJ23074
1	F13	sample	M-003112-03	DDR2
1	F14	sample	M-005320-01	LRRK1
1	F15	sample	M-006711-02	DGKA

1	F16	sample	M-005321-00	FLJ23356
1	F17	sample	M-006712-01	DGKB
1	F18	sample	M-004624-00	FLJ25006
1	F19	sample	M-006713-03	DGKD
1	F20	sample	M-004052-02	FLJ32685
1	F21	sample	M-011493-00	DGKE
1	F22	sample	M-006803-00	C9ORF98
1	F23	sample	M-006715-00	DGKG
1	F24	sample	M-005326-00	FLJ34389
1	G01	empty	empty	empty
1	G02	empty	empty	empty
1	G03	control		TRAPPC3
1	G04	control		CLSPN
1	G05	sample	M-004504-02	ADAM9
1	G06	sample	M-004943-03	CAMK2B
1	G07	sample	M-005303-02	ADCK1
1	G08	sample	M-004042-02	CAMK2D
1	G09	sample	M-005304-01	ADCK2
1	G10	sample	M-004536-01	CAMK2G
1	G11	sample	M-005305-01	ADCK4
1	G12	sample	M-004944-01	CAMK4
1	G13	sample	M-018919-00	ADCK5
1	G14	sample	M-004912-00	CAMKK1
1	G15	sample	M-004733-02	ADK
1	G16	sample	M-004842-01	CAMKK2
1	G17	sample	M-005419-00	ADRA1A
1	G18	sample	M-004395-00	CARD10
1	G19	sample	M-005420-01	ADRA1B
1	G20	sample	M-004397-00	CARD14
1	G21	sample	M-005426-01	ADRB2
1	G22	sample	M-005013-01	TNNI3K
1	G23	sample	M-004325-01	ADRBK1
1	G24	sample	M-006815-00	CARKL
1	H01	empty	empty	empty
1	H02	empty	empty	empty
1	H03	control		TRAPPC3
1	H04	control		CLSPN
1	H05	sample	M-006717-01	DGKI
1	H06	sample	M-007727-00	FLJ35107
1	H07	sample	M-005079-02	DGKQ
1	H08	sample	M-003136-02	FLT1
1	H09	sample	M-006718-00	DGKZ
1	H10	sample	M-003137-01	FLT3
1	H11	sample	M-006719-02	DGUOK
1	H12	sample	M-003138-01	FLT4

1	H13	sample	M-004949-00	DKFZP434C131
1	H14	sample	M-006724-00	FN3K
1	H15	sample	M-005313-01	DKFZp434C1418
1	H16	sample	M-006817-00	FN3KRP
1	H17	sample	M-006808-00	DKFZP586B1621
1	H18	sample	M-003008-01	FRAP1
1	H19	sample	M-025870-00	DKFZP761P0423
1	H20	sample	M-006691-00	FRDA
1	H21	sample	M-003113-01	STYK1
1	H22	sample	M-003139-01	FRK
1	H23	sample	M-009415-00	DLG1
1	H24	sample	M-007255-00	FUK
1	I01	empty	empty	empty
1	I02	empty	empty	empty
1	I03	control		CLSPN
1	I04	control		TRAPPC3
1	I05	sample	M-004326-00	ADRBK2
1	I06	sample	M-005311-01	CASK
1	I07	sample	M-005429-01	AGTR2
1	I08	sample	M-007831-00	CCL2
1	I09	sample	M-006811-02	AK1
1	I10	sample	M-007843-01	CCL4
1	I11	sample	M-006812-00	AK2
1	I12	sample	M-004686-01	CCRK
1	I13	sample	M-006700-01	AK3
1	I14	sample	M-003775-01	CD3E
1	I15	sample	M-006701-00	AK3L1
1	I16	sample	M-005234-01	CD4
1	I17	sample	M-004897-02	AK5
1	I18	sample	M-013630-02	CD7
1	I19	sample	M-007257-00	AK7
1	I20	sample	M-007732-00	CDADC1
1	I21	sample	M-011426-01	AKAP1
1	I22	sample	M-003224-03	CDC2
1	I23	sample	M-009277-01	AKAP11
1	I24	sample	M-004687-01	CDC2L1
1	J01	empty	empty	empty
1	J02	empty	empty	empty
1	J03	control		CLSPN
1	J04	control		TRAPPC3
1	J05	sample	M-011252-01	DLG2
1	J06	sample	M-020174-01	FYB
1	J07	sample	M-009462-01	DLG3
1	J08	sample	M-003140-03	FYN
1	J09	sample	M-007882-01	DLG4

1	J10	sample	M-005005-01	GAK
1	J11	sample	M-004637-00	DMPK
1	J12	sample	M-007728-01	GALK1
1	J13	sample	M-012251-00	DJC3
1	J14	sample	M-006725-00	GALK2
1	J15	sample	M-011254-00	DOK1
1	J16	sample	M-011663-00	GAP43
1	J17	sample	M-006720-00	DTYMK
1	J18	sample	M-010819-01	GCK
1	J19	sample	M-003484-02	DUSP1
1	J20	sample	M-007914-00	GFRA2
1	J21	sample	M-003965-01	DUSP10
1	J22	sample	M-006727-00	GK
1	J23	sample	M-003565-01	DUSP2
1	J24	sample	M-015091-01	GK2
1	K01	empty	empty	empty
1	K02	empty	empty	empty
1	K03	control		KIF11
1	K04	control		CASP1
1	K05	sample	M-008868-01	AKAP13
1	K06	sample	M-004026-01	CDC2L2
1	K07	sample	M-009765-01	AKAP3
1	K08	sample	M-004688-00	CDC2L5
1	K09	sample	M-008442-00	AKAP4
1	K10	sample	M-003814-02	CDC42BPA
1	K11	sample	M-011954-00	AKAP5
1	K12	sample	M-004075-01	CDC42BPB
1	K13	sample	M-008497-01	AKAP6
1	K14	sample	M-003234-02	CDC7
1	K15	sample	M-013371-00	AKAP7
1	K16	sample	M-003235-04	CDK10
1	K17	sample	M-009656-01	AKAP8
1	K18	sample	M-004689-00	CDK11
1	K19	sample	M-003000-01	AKT1
1	K20	sample	M-003236-04	CDK2
1	K21	sample	M-003001-01	AKT2
1	K22	sample	M-003237-01	CDK3
1	K23	sample	M-003002-01	AKT3
1	K24	sample	M-003238-02	CDK4
1	L01	empty	empty	empty
1	L02	empty	empty	empty
1	L03	control		KIF11
1	L04	control		CASP1
1	L05	sample	M-004517-00	DUSP22
1	L06	sample	M-019100-00	GMFB

1	L07	sample	M-003963-02	DUSP4
1	L08	sample	M-019878-01	GMFG
1	L09	sample	M-003566-01	DUSP5
1	L10	sample	M-006729-00	GNE
1	L11	sample	M-003964-01	DUSP6
1	L12	sample	M-004625-00	GRK4
1	L13	sample	M-003567-00	DUSP7
1	L14	sample	M-004626-00	GRK5
1	L15	sample	M-003568-00	DUSP8
1	L16	sample	M-004627-01	GRK6
1	L17	sample	M-004805-00	DYRK1A
1	L18	sample	M-004628-00	GRK7
1	L19	sample	M-004806-01	DYRK1B
1	L20	sample	M-005327-00	GSG2
1	L21	sample	M-004730-02	DYRK2
1	L22	sample	M-003009-01	GSK3A
1	L23	sample	M-004731-00	DYRK3
1	L24	sample	M-003010-03	GSK3B
1	M01	empty	empty	empty
1	M02	empty	empty	empty
1	M03	control		KIF23
1	M04	control		RLUC
1	M05	sample	M-003103-02	ALK
1	M06	sample	M-003239-01	CDK5
1	M07	sample	M-004929-01	ACVR1C
1	M08	sample	M-008988-00	CDK5R1
1	M09	sample	M-005306-01	ALS2CR2
1	M10	sample	M-008885-00	CDK5R2
1	M11	sample	M-004685-01	ALS2CR7
1	M12	sample	M-013297-01	CDK5RAP1
1	M13	sample	M-005307-01	AMHR2
1	M14	sample	M-012957-00	CDK5RAP3
1	M15	sample	M-007803-00	ANGPT4
1	M16	sample	M-003240-02	CDK6
1	M17	sample	M-004930-01	ANKK1
1	M18	sample	M-003241-01	CDK7
1	M19	sample	M-005308-02	ANKRD3
1	M20	sample	M-003242-02	CDK8
1	M21	sample	M-007687-01	APEG1
1	M22	sample	M-003243-02	CDK9
1	M23	sample	M-005138-00	APPL
1	M24	sample	M-004323-01	CDKL1
1	N01	empty	empty	empty
1	N02	empty	empty	empty
1	N03	control		KIF23

1	N04	control		RLUC
1	N05	sample	M-004732-01	DYRK4
1	N06	sample	M-010924-00	GTF2H1
1	N07	sample	M-017723-01	EDN2
1	N08	sample	M-005328-00	GUCY2C
1	N09	sample	M-004950-00	EEF2K
1	N10	sample	M-005329-01	GUCY2D
1	N11	sample	M-003114-01	EGFR
1	N12	sample	M-004515-01	GUCY2F
1	N13	sample	M-004883-01	EIF2AK3
1	N14	sample	M-006734-01	GUK1
1	N15	sample	M-005314-00	EIF2AK4
1	N16	sample	M-005006-00	HSPB8
1	N17	sample	M-006721-01	EKI1
1	N18	sample	M-005330-02	HAK
1	N19	sample	M-007676-00	RAPGEF3
1	N20	sample	M-003141-02	HCK
1	N21	sample	M-003115-02	EPHA1
1	N22	sample	M-004809-02	HIPK1
1	N23	sample	M-003116-01	EPHA2
1	N24	sample	M-003266-03	HIPK2
1	O01	empty	empty	empty
1	O02	empty	empty	empty
1	O03	control		PLK1
1	O04	control		UBC
1	O05	sample	M-003563-02	ARAF1
1	O06	sample	M-004797-00	CDKL2
1	O07	sample	M-004931-00	ARK5
1	O08	sample	M-004798-00	CDKL3
1	O09	sample	M-004165-01	ASK
1	O10	sample	M-004799-02	CDKL5
1	O11	sample	M-013023-00	ASP
1	O12	sample	M-003471-00	CDKN1A
1	O13	sample	M-003201-02	ATM
1	O14	sample	M-003472-00	CDKN1B
1	O15	sample	M-003202-04	ATR
1	O16	sample	M-003244-03	CDKN1C
1	O17	sample	M-003326-02	AURKB
1	O18	sample	M-003245-02	CDKN2B
1	O19	sample	M-019573-01	AURKC
1	O20	sample	M-003246-01	CDKN2C
1	O21	sample	M-003631-01	AVPR1A
1	O22	sample	M-003247-02	CDKN2D
1	O23	sample	M-005431-00	AVPR1B
1	O24	sample	M-003879-00	CDKN3

1	P01	empty	empty	empty
1	P02	empty	empty	empty
1	P03	control		PLK1
1	P04	control		UBC
1	P05	sample	M-003117-02	EPHA3
1	P06	sample	M-004810-00	HIPK3
1	P07	sample	M-003118-01	EPHA4
1	P08	sample	M-004808-02	HIPK4
1	P09	sample	M-005315-02	EPHA5
1	P10	sample	M-006820-01	HK1
1	P11	sample	M-003119-01	EPHA7
1	P12	sample	M-006735-01	HK2
1	P13	sample	M-003120-02	EPHA8
1	P14	sample	M-006736-00	HK3
1	P15	sample	M-003121-01	EPHB1
1	P16	sample	M-005007-00	HRI
1	P17	sample	M-003122-01	EPHB2
1	P18	sample	M-004618-00	STK32B
1	P19	sample	M-003123-02	EPHB3
1	P20	sample	M-007691-01	HSMDPKIN
1	P21	sample	M-003124-01	EPHB4
1	P22	sample	M-004214-01	HUNK
1	P23	sample	M-003125-01	EPHB6
1	P24	sample	M-011927-00	ITGB1BP1
2	A01	empty	empty	empty
2	A02	empty	empty	empty
2	A03	control		UBC
2	A04	control		PLK1
2	A05	sample	M-004811-01	ICK
2	A06	sample	M-003789-02	MAP3K4
2	A07	sample	M-003012-04	IGF1R
2	A08	sample	M-003584-02	MAP3K5
2	A09	sample	M-006737-01	IHPK1
2	A10	sample	M-003969-00	MAP3K6
2	A11	sample	M-006738-00	IHPK2
2	A12	sample	M-003790-05	MAP3K7
2	A13	sample	M-006739-00	IHPK3
2	A14	sample	M-003511-03	MAP3K8
2	A15	sample	M-009371-00	IKBKAP
2	A16	sample	M-003585-01	MAP3K9
2	A17	sample	M-003503-00	IKBKB
2	A18	sample	M-003586-01	MAP4K1
2	A19	sample	M-003723-02	IKBKE
2	A20	sample	M-003587-01	MAP4K2
2	A21	sample	M-007967-00	IL2

2	A22	sample	M-003588-01	MAP4K3
2	A23	sample	M-004499-00	ILK
2	A24	sample	M-003971-02	MAP4K4
2	B01	empty	empty	empty
2	B02	empty	empty	empty
2	B03	control		UBC
2	B04	control		PLK1
2	B05	sample	M-004869-01	NEK9
2	B06	sample	M-006779-02	PIP5K2B
2	B07	sample	M-004763-01	NLK
2	B08	sample	M-004535-00	PIP5K2C
2	B09	sample	M-006821-01	NME1
2	B10	sample	M-014699-00	PITPNM3
2	B11	sample	M-005102-01	NME2
2	B12	sample	M-004615-00	STK32C
2	B13	sample	M-006753-00	NME3
2	B14	sample	M-012321-00	PKIA
2	B15	sample	M-006494-00	NME4
2	B16	sample	M-008224-01	PKIB
2	B17	sample	M-006754-00	NME5
2	B18	sample	M-006780-00	PKLR
2	B19	sample	M-006755-01	NME6
2	B20	sample	M-006781-01	PKM2
2	B21	sample	M-006756-02	NME7
2	B22	sample	M-005026-02	PKMYT1
2	B23	sample	M-005354-00	NPR1
2	B24	sample	M-004647-00	PKN3
2	C01	empty	empty	empty
2	C02	empty	empty	empty
2	C03	control		RLUC
2	C04	control		KIF23
2	C05	sample	M-010260-00	ILKAP
2	C06	sample	M-003589-02	MAP4K5
2	C07	sample	M-006740-01	IMPK
2	C08	sample	M-003555-02	MAPK1
2	C09	sample	M-003014-01	INSR
2	C10	sample	M-004324-00	MAPK10
2	C11	sample	M-005332-00	INSRR
2	C12	sample	M-003972-03	MAPK11
2	C13	sample	M-004760-02	IRAK1
2	C14	sample	M-003590-00	MAPK12
2	C15	sample	M-004761-00	IRAK2
2	C16	sample	M-003591-02	MAPK13
2	C17	sample	M-004762-00	IRAK3
2	C18	sample	M-003512-05	MAPK14

2	C19	sample	M-003015-01	IRS1
2	C20	sample	M-003592-02	MAPK3
2	C21	sample	M-003144-02	ITK
2	C22	sample	M-003593-02	MAPK4
2	C23	sample	M-006741-00	ITPK1
2	C24	sample	M-003594-01	MAPK6
2	D01	empty	empty	empty
2	D02	empty	empty	empty
2	D03	control		RLUC
2	D04	control		KIF23
2	D05	sample	M-005355-01	NPR2
2	D06	sample	M-003290-01	PLK1
2	D07	sample	M-005356-01	NRBP
2	D08	sample	M-010904-00	EXOSC10
2	D09	sample	M-026286-00	NRG3
2	D10	sample	M-006782-00	PMVK
2	D11	sample	M-003159-01	NTRK1
2	D12	sample	M-006783-02	PNKP
2	D13	sample	M-003160-01	NTRK2
2	D14	sample	M-012745-00	PPP1R1B
2	D15	sample	M-003161-01	NTRK3
2	D16	sample	M-003598-00	PPP2CA
2	D17	sample	M-007733-00	NYD-SP25
2	D18	sample	M-003599-02	PPP2CB
2	D19	sample	M-004870-01	OSR1
2	D20	sample	M-008486-01	PPP4C
2	D21	sample	M-007734-01	P15RS
2	D22	sample	M-005027-01	PRKAA1
2	D23	sample	M-005357-00	PACE-1
2	D24	sample	M-005361-01	PRKAA2
2	E01	empty	empty	empty
2	E02	empty	empty	empty
2	E03	control		CASP1
2	E04	control		KIF11
2	E05	sample	M-006742-01	ITPKA
2	E06	sample	M-003513-02	MAPK7
2	E07	sample	M-006743-02	ITPKB
2	E08	sample	M-003514-01	MAPK8
2	E09	sample	M-006744-01	ITPKC
2	E10	sample	M-003595-00	MAPK8IP1
2	E11	sample	M-003145-01	JAK1
2	E12	sample	M-012462-00	MAPK8IP2
2	E13	sample	M-003146-02	JAK2
2	E14	sample	M-003596-01	MAPK8IP3
2	E15	sample	M-003147-01	JAK3

2	E16	sample	M-003505-02	MAPK9
2	E17	sample	M-004844-01	JIK
2	E18	sample	M-003516-02	MAPKAPK2
2	E19	sample	M-003148-01	KDR
2	E20	sample	M-005014-01	MAPKAPK3
2	E21	sample	M-006745-02	KHK
2	E22	sample	M-005015-00	MAPKAPK5
2	E23	sample	M-004542-02	TNIK
2	E24	sample	M-004259-02	MARK1
2	F01	empty	empty	empty
2	F02	empty	empty	empty
2	F03	control		CASP1
2	F04	control		KIF11
2	F05	sample	M-007735-00	PACSIN1
2	F06	sample	M-004649-00	PRKACA
2	F07	sample	M-012956-01	PAG
2	F08	sample	M-004650-00	PRKACB
2	F09	sample	M-003521-03	PAK1
2	F10	sample	M-004651-02	PRKACG
2	F11	sample	M-003597-02	PAK2
2	F12	sample	M-009056-00	PRKAG1
2	F13	sample	M-003614-00	PAK3
2	F14	sample	M-009859-01	PRKAG3
2	F15	sample	M-003615-02	PAK4
2	F16	sample	M-007670-01	PRKAR1A
2	F17	sample	M-004338-02	PAK6
2	F18	sample	M-007671-00	PRKAR2A
2	F19	sample	M-003973-02	PAK7
2	F20	sample	M-007673-00	PRKAR2B
2	F21	sample	M-004057-02	PANK1
2	F22	sample	M-003523-03	PRKCA
2	F23	sample	M-006758-00	PANK3
2	F24	sample	M-020124-00	PRKCABP
2	G01	empty	empty	empty
2	G02	empty	empty	empty
2	G03	control		TRAPPC3
2	G04	control		CLSPN
2	G05	sample	M-004046-00	MAST3
2	G06	sample	M-004260-01	MARK2
2	G07	sample	M-004779-02	KIAA0999
2	G08	sample	M-003517-03	MARK3
2	G09	sample	M-004846-01	KIAA1361
2	G10	sample	M-005345-02	MARK4
2	G11	sample	M-023172-01	KIAA1399
2	G12	sample	M-004633-00	MAST2

2	G13	sample	M-005336-00	KIAA1639
2	G14	sample	M-003154-03	MATK
2	G15	sample	M-005337-00	KIAA1765
2	G16	sample	M-021396-00	MBIP
2	G17	sample	M-004063-00	KIAA1804
2	G18	sample	M-004029-01	MELK
2	G19	sample	M-004619-01	KIAA1811
2	G20	sample	M-003155-01	MERTK
2	G21	sample	M-005338-01	LMTK3
2	G22	sample	M-003156-02	MET
2	G23	sample	M-004963-00	KIF13B
2	G24	sample	M-005346-00	MGC16169
2	H01	empty	empty	empty
2	H02	empty	empty	empty
2	H03	control		TRAPPC3
2	H04	control		CLSPN
2	H05	sample	M-006759-00	PANK4
2	H06	sample	M-003758-04	PRKCB1
2	H07	sample	M-007736-01	PAPSS1
2	H08	sample	M-003524-01	PRKCD
2	H09	sample	M-006760-01	PAPSS2
2	H10	sample	M-004653-00	PRKCE
2	H11	sample	M-005018-01	PASK
2	H12	sample	M-004654-01	PRKCG
2	H13	sample	M-006796-00	PCK1
2	H14	sample	M-004655-01	PRKCH
2	H15	sample	M-006797-01	PCK2
2	H16	sample	M-004656-01	PRKCI
2	H17	sample	M-004313-00	PCTK1
2	H18	sample	M-004175-02	PRKCL1
2	H19	sample	M-004835-01	PCTK2
2	H20	sample	M-004612-02	PRKCL2
2	H21	sample	M-004836-02	PCTK3
2	H22	sample	M-005028-00	PRKCM
2	H23	sample	M-003162-03	PDGFRA
2	H24	sample	M-005029-01	PRKCN
2	I01	empty	empty	empty
2	I02	empty	empty	empty
2	I03	control		CLSPN
2	I04	control		TRAPPC3
2	I05	sample	M-003981-01	KIS
2	I06	sample	M-004634-00	STK32A
2	I07	sample	M-003150-01	KIT
2	I08	sample	M-004783-00	MGC26597
2	I09	sample	M-003149-01	LMTK2

2	I10	sample	M-004681-01	CSNK1A1L
2	I11	sample	M-005322-01	KSR2
2	I12	sample	M-005016-01	MGC42105
2	I13	sample	M-005009-01	LAK
2	I14	sample	M-005347-00	C9ORF96
2	I15	sample	M-004632-00	LATS1
2	I16	sample	M-005017-01	MGC45428
2	I17	sample	M-003865-00	LATS2
2	I18	sample	M-008770-00	PIP5KL1
2	I19	sample	M-003151-01	LCK
2	I20	sample	M-005348-00	MGC4796
2	I21	sample	M-012120-00	LCP2
2	I22	sample	M-007684-01	MGC5601
2	I23	sample	M-006930-00	LIM
2	I24	sample	M-005349-01	MGC8407
2	J01	empty	empty	empty
2	J02	empty	empty	empty
2	J03	control		CLSPN
2	J04	control		TRAPPC3
2	J05	sample	M-003163-02	PDGFRB
2	J06	sample	M-003525-01	PRKCQ
2	J07	sample	M-005019-00	PDK1
2	J08	sample	M-010618-00	PRKCSH
2	J09	sample	M-005020-00	PDK2
2	J10	sample	M-003526-02	PRKCZ
2	J11	sample	M-005021-01	PDK3
2	J12	sample	M-004197-01	PRKD2
2	J13	sample	M-019425-00	PDK4
2	J14	sample	M-005030-01	PRKDC
2	J15	sample	M-003017-01	PDPK1
2	J16	sample	M-004658-02	PRKG1
2	J17	sample	M-005070-02	PDXK
2	J18	sample	M-004659-00	PRKG2
2	J19	sample	M-006761-00	PFKFB1
2	J20	sample	M-003527-00	PRKR
2	J21	sample	M-006762-01	PFKFB2
2	J22	sample	M-006426-00	PRKRA
2	J23	sample	M-006763-00	PFKFB3
2	J24	sample	M-005362-00	PRKWNK1
2	K01	empty	empty	empty
2	K02	empty	empty	empty
2	K03	control		KIF11
2	K04	control		CASP1
2	K05	sample	M-007730-01	LIMK1
2	K06	sample	M-005350-01	MIDORI

2	K07	sample	M-003311-02	LIMK2
2	K08	sample	M-004861-02	MINK
2	K09	sample	M-015901-01	LOC115704
2	K10	sample	M-004879-01	MKNK1
2	K11	sample	M-005011-01	LOC149420
2	K12	sample	M-004908-01	MKNK2
2	K13	sample	M-006804-00	PRPS1L1
2	K14	sample	M-003859-02	MOS
2	K15	sample	M-005340-01	LOC340371
2	K16	sample	M-010252-00	MPP1
2	K17	sample	M-005342-02	LOC91807
2	K18	sample	M-009729-00	MPP2
2	K19	sample	M-003152-01	LTK
2	K20	sample	M-010612-01	MPP3
2	K21	sample	M-005343-01	LYK5
2	K22	sample	M-015738-01	MPZL1
2	K23	sample	M-003153-03	LYN
2	K24	sample	M-020064-00	MRC2
2	L01	empty	empty	empty
2	L02	empty	empty	empty
2	L03	control		KIF11
2	L04	control		CASP1
2	L05	sample	M-006764-00	PFKFB4
2	L06	sample	M-005363-01	PRKWNK2
2	L07	sample	M-006822-00	PFKL
2	L08	sample	M-005364-02	PRKWNK3
2	L09	sample	M-006765-01	PFKM
2	L10	sample	M-005031-01	PRKWNK4
2	L11	sample	M-010253-01	PFKP
2	L12	sample	M-004660-01	PRKX
2	L13	sample	M-004837-01	PFTK1
2	L14	sample	M-004661-02	PRKY
2	L15	sample	M-006767-01	PGK1
2	L16	sample	M-004074-03	PRPF4B
2	L17	sample	M-006768-01	PGK2
2	L18	sample	M-006784-00	PRPS1
2	L19	sample	M-019682-00	PHKA1
2	L20	sample	M-004877-01	PRPS2
2	L21	sample	M-007669-00	PHKA2
2	L22	sample	M-006794-01	PRPSAP1
2	L23	sample	M-005023-01	PHKG1
2	L24	sample	M-006795-01	PRPSAP2
2	M01	empty	empty	empty
2	M02	empty	empty	empty
2	M03	control		KIF23

2	M04	control		RLUC
2	M05	sample	M-020068-00	SMAD7
2	M06	sample	M-003157-02	MST1R
2	M07	sample	M-009453-01	MAGI-3
2	M08	sample	M-003753-01	MST4
2	M09	sample	M-004813-01	MAK
2	M10	sample	M-003158-01	MUSK
2	M11	sample	M-005936-01	MALT1
2	M12	sample	M-006748-00	MVD
2	M13	sample	M-003571-01	MAP2K1
2	M14	sample	M-006749-00	MVK
2	M15	sample	M-003572-02	MAP2K1IP1
2	M16	sample	M-005351-04	MYLK
2	M17	sample	M-003573-03	MAP2K2
2	M18	sample	M-005352-02	MYLK2
2	M19	sample	M-003509-01	MAP2K3
2	M20	sample	M-004862-00	MYO3A
2	M21	sample	M-003574-02	MAP2K4
2	M22	sample	M-004863-00	MYO3B
2	M23	sample	M-003966-03	MAP2K5
2	M24	sample	M-006750-00	GK
2	N01	empty	empty	empty
2	N02	empty	empty	empty
2	N03	control		KIF23
2	N04	control		RLUC
2	N05	sample	M-004881-00	PHKG2
2	N06	sample	M-004171-03	TAO1
2	N07	sample	M-006769-01	PI4K2B
2	N08	sample	M-005365-00	PSKH1
2	N09	sample	M-006770-01	PI4KII
2	N10	sample	M-005366-00	PSKH2
2	N11	sample	M-006771-00	PIK3C2A
2	N12	sample	M-003164-02	PTK2
2	N13	sample	M-006772-01	PIK3C2B
2	N14	sample	M-003165-03	PTK2B
2	N15	sample	M-006773-00	PIK3C2G
2	N16	sample	M-003166-01	PTK6
2	N17	sample	M-003018-01	PIK3CA
2	N18	sample	M-003167-02	PTK7
2	N19	sample	M-003019-02	PIK3CB
2	N20	sample	M-003168-03	PTK9
2	N21	sample	M-005274-02	PIK3CG
2	N22	sample	M-003169-01	PTK9L
2	N23	sample	M-003020-02	PIK3R1
2	N24	sample	M-003600-01	PTPN5

2	O01	empty	empty	empty
2	O02	empty	empty	empty
2	O03	control		PLK1
2	O04	control		UBC
2	O05	sample	M-003967-00	MAP2K6
2	O06	sample	M-015419-00	NBEA
2	O07	sample	M-004016-01	MAP2K7
2	O08	sample	M-006751-00	COASY
2	O09	sample	M-003575-01	MAP3K1
2	O10	sample	M-004864-00	NEK1
2	O11	sample	M-003576-01	MAP3K10
2	O12	sample	M-004865-01	NEK11
2	O13	sample	M-003577-02	MAP3K11
2	O14	sample	M-004090-03	NEK2
2	O15	sample	M-003312-02	MAP3K12
2	O16	sample	M-004867-01	NEK3
2	O17	sample	M-003579-01	MAP3K13
2	O18	sample	M-003519-01	NEK4
2	O19	sample	M-003580-03	MAP3K14
2	O20	sample	M-004166-01	NEK6
2	O21	sample	M-003582-01	MAP3K2
2	O22	sample	M-003795-02	NEK7
2	O23	sample	M-003301-02	MAP3K3
2	O24	sample	M-004866-00	NEK8
2	P01	empty	empty	empty
2	P02	empty	empty	empty
2	P03	control		PLK1
2	P04	control		UBC
2	P05	sample	M-003021-01	PIK3R2
2	P06	sample	M-008069-00	PTPRG
2	P07	sample	M-019546-00	PIK3R3
2	P08	sample	M-008476-01	PTPRJ
2	P09	sample	M-005025-01	PIK3R4
2	P10	sample	M-004017-01	PTPRR
2	P11	sample	M-006776-03	PIK4CA
2	P12	sample	M-008072-01	PTPRT
2	P13	sample	M-006777-02	PIK4CB
2	P14	sample	M-005367-00	PXK
2	P15	sample	M-003923-00	PIM1
2	P16	sample	M-006785-00	PYCS
2	P17	sample	M-005359-00	PIM2
2	P18	sample	M-003560-02	RAC1
2	P19	sample	M-004030-01	PINK1
2	P20	sample	M-003601-00	RAF1
2	P21	sample	M-004780-02	PIP5K1A

2	P22	sample	M-004838-01	RAGE
2	P23	sample	M-006778-00	PIP5K2A
2	P24	sample	M-024516-01	RASGRF2
3	A01	empty	empty	empty
3	A02	empty	empty	empty
3	A03	control		UBC
3	A04	control		PLK1
3	A05	sample	M-003176-03	SYK
3	A06	sample	M-006786-00	RBKS
3	A07	sample	M-005041-01	TAF1
3	A08	sample	M-003170-01	RET
3	A09	sample	M-005385-02	TAF1L
3	A10	sample	M-006552-00	RFP
3	A11	sample	M-004171-03	TAO1
3	A12	sample	M-004662-00	GRK1
3	A13	sample	M-003788-02	TBK1
3	A14	sample	M-005368-01	RIOK1
3	A15	sample	M-003177-02	TEC
3	A16	sample	M-005040-01	RIOK3
3	A17	sample	M-003178-02	TEK
3	A18	sample	M-004445-01	RIPK1
3	A19	sample	M-005043-00	TESK1
3	A20	sample	M-003602-00	RIPK2
3	A21	sample	M-005044-00	TESK2
3	A22	sample	M-003534-00	RIPK3
3	A23	sample	M-005386-01	TEX14
3	A24	sample	M-005032-01	RSEL
3	B01	empty	empty	empty
3	B02	empty	empty	empty
3	B03	control		UBC
3	B04	control		PLK1
3	B05	empty	empty	empty
3	B06	empty	empty	empty
3	B07	empty	empty	empty
3	B08	empty	empty	empty
3	B09	empty	empty	empty
3	B10	empty	empty	empty
3	B11	empty	empty	empty
3	B12	empty	empty	empty
3	B13	empty	empty	empty
3	B14	empty	empty	empty
3	B15	empty	empty	empty
3	B16	empty	empty	empty
3	B17	empty	empty	empty
3	B18	empty	empty	empty

3	B19	empty	empty	empty
3	B20	empty	empty	empty
3	B21	empty	empty	empty
3	B22	empty	empty	empty
3	B23	empty	empty	empty
3	B24	empty	empty	empty
3	C01	empty	empty	empty
3	C02	empty	empty	empty
3	C03	control		RLUC
3	C04	control		KIF23
3	C05	sample	M-003929-01	TGFBR1
3	C06	sample	M-003536-01	ROCK1
3	C07	sample	M-003930-01	TGFBR2
3	C08	sample	M-004610-01	ROCK2
3	C09	sample	M-003179-01	TIE
3	C10	sample	M-003171-01	ROR1
3	C11	sample	M-009932-01	TJP2
3	C12	sample	M-003172-01	ROR2
3	C13	sample	M-006787-00	TK1
3	C14	sample	M-003173-01	ROS1
3	C15	sample	M-006788-02	TK2
3	C16	sample	M-012350-01	RP2
3	C17	sample	M-004174-00	TLK1
3	C18	sample	M-003025-03	RPS6KA1
3	C19	sample	M-005389-02	TLK2
3	C20	sample	M-004663-01	RPS6KA2
3	C21	sample	M-008086-00	TLR1
3	C22	sample	M-003026-01	RPS6KA3
3	C23	sample	M-007745-00	TLR3
3	C24	sample	M-004664-00	RPS6KA4
3	D01	empty	empty	empty
3	D02	empty	empty	empty
3	D03	control		RLUC
3	D04	control		KIF23
3	D05	empty	empty	empty
3	D06	empty	empty	empty
3	D07	empty	empty	empty
3	D08	empty	empty	empty
3	D09	empty	empty	empty
3	D10	empty	empty	empty
3	D11	empty	empty	empty
3	D12	empty	empty	empty
3	D13	empty	empty	empty
3	D14	empty	empty	empty
3	D15	empty	empty	empty

3	D16	empty	empty	empty
3	D17	empty	empty	empty
3	D18	empty	empty	empty
3	D19	empty	empty	empty
3	D20	empty	empty	empty
3	D21	empty	empty	empty
3	D22	empty	empty	empty
3	D23	empty	empty	empty
3	D24	empty	empty	empty
3	E01	empty	empty	empty
3	E02	empty	empty	empty
3	E03	control		CASP1
3	E04	control		KIF11
3	E05	sample	M-008088-00	TLR4
3	E06	sample	M-004665-01	RPS6KA5
3	E07	sample	M-005156-01	TLR6
3	E08	sample	M-004670-00	RPS6KA6
3	E09	sample	M-008090-01	TNFRSF10A
3	E10	sample	M-003616-02	RPS6KB1
3	E11	sample	M-003180-02	TNK1
3	E12	sample	M-004671-00	RPS6KB2
3	E13	sample	M-005390-00	TOPK
3	E14	sample	M-005371-01	RPS6KC1
3	E15	sample	M-006789-00	TPK1
3	E16	sample	M-005372-00	RPS6KL1
3	E17	sample	M-005045-00	KALRN
3	E18	sample	M-003174-02	RYK
3	E19	sample	M-005391-01	TRIB2
3	E20	sample	M-004672-01	SAST
3	E21	sample	M-020821-00	TRIM
3	E22	sample	M-011505-01	SCAP1
3	E23	sample	M-005047-00	TRIO
3	E24	sample	M-005373-00	SCYL1
3	F01	empty	empty	empty
3	F02	empty	empty	empty
3	F03	control		CASP1
3	F04	control		KIF11
3	F05	empty	empty	empty
3	F06	empty	empty	empty
3	F07	empty	empty	empty
3	F08	empty	empty	empty
3	F09	empty	empty	empty
3	F10	empty	empty	empty
3	F11	empty	empty	empty
3	F12	empty	empty	empty

3	F13	empty	empty	empty
3	F14	empty	empty	empty
3	F15	empty	empty	empty
3	F16	empty	empty	empty
3	F17	empty	empty	empty
3	F18	empty	empty	empty
3	F19	empty	empty	empty
3	F20	empty	empty	empty
3	F21	empty	empty	empty
3	F22	empty	empty	empty
3	F23	empty	empty	empty
3	F24	empty	empty	empty
3	G01	empty	empty	empty
3	G02	empty	empty	empty
3	G03	control		TRAPPC3
3	G04	control		CLSPN
3	G05	sample	M-005048-01	TRPM6
3	G06	sample	M-007737-00	SEPHS1
3	G07	sample	M-005393-02	TRPM7
3	G08	sample	M-003027-04	SGK
3	G09	sample	M-013077-01	TSKS
3	G10	sample	M-004673-01	SGK2
3	G11	sample	M-004680-01	TTBK1
3	G12	sample	M-004162-00	SGKL
3	G13	sample	M-004682-00	TTBK2
3	G14	sample	M-018841-00	SHC1
3	G15	sample	M-004105-00	TTK
3	G16	sample	M-004778-02	SIK2
3	G17	sample	M-005395-00	TTN
3	G18	sample	M-003850-02	SLK
3	G19	sample	M-003181-01	TXK
3	G20	sample	M-005033-00	SMG1
3	G21	sample	M-006791-00	TXNDC3
3	G22	sample	M-005374-01	SRK
3	G23	sample	M-003182-01	TYK2
3	G24	sample	M-003959-04	SNF1LK
3	H01	empty	empty	empty
3	H02	empty	empty	empty
3	H03	control		TRAPPC3
3	H04	control		CLSPN
3	H05	empty	empty	empty
3	H06	empty	empty	empty
3	H07	empty	empty	empty
3	H08	empty	empty	empty
3	H09	empty	empty	empty

3	H10	empty	empty	empty
3	H11	empty	empty	empty
3	H12	empty	empty	empty
3	H13	empty	empty	empty
3	H14	empty	empty	empty
3	H15	empty	empty	empty
3	H16	empty	empty	empty
3	H17	empty	empty	empty
3	H18	empty	empty	empty
3	H19	empty	empty	empty
3	H20	empty	empty	empty
3	H21	empty	empty	empty
3	H22	empty	empty	empty
3	H23	empty	empty	empty
3	H24	empty	empty	empty
3	I01	empty	empty	empty
3	I02	empty	empty	empty
3	I03	control		CLSPN
3	I04	control		TRAPPC3
3	I05	sample	M-003183-02	TYRO3
3	I06	sample	M-003325-03	PLK2
3	I07	sample	M-004062-01	UCK1
3	I08	sample	M-004322-00	SNRK
3	I09	sample	M-007739-02	UGP2
3	I10	sample	M-011511-01	SOCS1
3	I11	sample	M-005049-00	ULK1
3	I12	sample	M-017374-00	SOCS5
3	I13	sample	M-005396-01	ULK2
3	I14	sample	M-007677-01	SPA17
3	I15	sample	M-004059-00	UMP-CMPK
3	I16	sample	M-020826-00	SPEC2
3	I17	sample	M-005077-00	UMPK
3	I18	sample	M-004172-02	SPHK1
3	I19	sample	M-006792-00	URKL1
3	I20	sample	M-004831-00	SPHK2
3	I21	sample	M-004683-01	VRK1
3	I22	sample	M-007738-00	SEPHS2
3	I23	sample	M-004684-01	VRK2
3	I24	sample	M-010230-00	SQSTM1
3	J01	empty	empty	empty
3	J02	empty	empty	empty
3	J03	control		CLSPN
3	J04	control		TRAPPC3
3	J05	empty	empty	empty
3	J06	empty	empty	empty

3	J07	empty	empty	empty
3	J08	empty	empty	empty
3	J09	empty	empty	empty
3	J10	empty	empty	empty
3	J11	empty	empty	empty
3	J12	empty	empty	empty
3	J13	empty	empty	empty
3	J14	empty	empty	empty
3	J15	empty	empty	empty
3	J16	empty	empty	empty
3	J17	empty	empty	empty
3	J18	empty	empty	empty
3	J19	empty	empty	empty
3	J20	empty	empty	empty
3	J21	empty	empty	empty
3	J22	empty	empty	empty
3	J23	empty	empty	empty
3	J24	empty	empty	empty
3	K01	empty	empty	empty
3	K02	empty	empty	empty
3	K03	control		KIF11
3	K04	control		CASP1
3	K05	sample	M-005397-01	VRK3
3	K06	sample	M-003175-03	SRC
3	K07	sample	M-005050-00	WEE1
3	K08	sample	M-005376-00	SRMS
3	K09	sample	M-012386-00	WIF1
3	K10	sample	M-003982-02	SRPK1
3	K11	sample	M-006793-00	XYLB
3	K12	sample	M-004839-01	SRPK2
3	K13	sample	M-003184-02	YES1
3	K14	sample	M-007290-00	ABI1
3	K15	sample	M-010626-00	YWHAH
3	K16	sample	M-005034-00	SSTK
3	K17	sample	M-012329-00	YWHAQ
3	K18	sample	M-004168-01	STK10
3	K19	sample	M-005068-00	ZAK
3	K20	sample	M-005035-01	STK11
3	K21	sample	M-005398-03	ZAP70
3	K22	sample	M-004054-00	STK16
3	K23	empty	empty	empty
3	K24	sample	M-005377-00	STK17A
3	L01	empty	empty	empty
3	L02	empty	empty	empty
3	L03	control		KIF11

3	L04	control		CASP1
3	L05	empty	empty	empty
3	L06	empty	empty	empty
3	L07	empty	empty	empty
3	L08	empty	empty	empty
3	L09	empty	empty	empty
3	L10	empty	empty	empty
3	L11	empty	empty	empty
3	L12	empty	empty	empty
3	L13	empty	empty	empty
3	L14	empty	empty	empty
3	L15	empty	empty	empty
3	L16	empty	empty	empty
3	L17	empty	empty	empty
3	L18	empty	empty	empty
3	L19	empty	empty	empty
3	L20	empty	empty	empty
3	L21	empty	empty	empty
3	L22	empty	empty	empty
3	L23	empty	empty	empty
3	L24	empty	empty	empty
3	M01	empty	empty	empty
3	M02	empty	empty	empty
3	M03	control		KIF23
3	M04	control		RLUC
3	M05	empty	empty	empty
3	M06	sample	M-004051-01	STK17B
3	M07	empty	empty	empty
3	M08	sample	M-005036-01	PLK4
3	M09	empty	empty	empty
3	M10	sample	M-005378-00	STK19
3	M11	empty	empty	empty
3	M12	sample	M-005379-01	STK22B
3	M13	empty	empty	empty
3	M14	sample	M-004050-01	STK22C
3	M15	empty	empty	empty
3	M16	sample	M-005038-02	STK22D
3	M17	empty	empty	empty
3	M18	sample	M-004840-02	STK23
3	M19	empty	empty	empty
3	M20	sample	M-004872-02	STK24
3	M21	empty	empty	empty
3	M22	sample	M-004873-00	STK25
3	M23	empty	empty	empty
3	M24	sample	M-005381-02	STK29

3	N01	empty	empty	empty
3	N02	empty	empty	empty
3	N03	control		KIF23
3	N04	control		RLUC
3	N05	empty	empty	empty
3	N06	empty	empty	empty
3	N07	empty	empty	empty
3	N08	empty	empty	empty
3	N09	empty	empty	empty
3	N10	empty	empty	empty
3	N11	empty	empty	empty
3	N12	empty	empty	empty
3	N13	empty	empty	empty
3	N14	empty	empty	empty
3	N15	empty	empty	empty
3	N16	empty	empty	empty
3	N17	empty	empty	empty
3	N18	empty	empty	empty
3	N19	empty	empty	empty
3	N20	empty	empty	empty
3	N21	empty	empty	empty
3	N22	empty	empty	empty
3	N23	empty	empty	empty
3	N24	empty	empty	empty
3	O01	empty	empty	empty
3	O02	empty	empty	empty
3	O03	control		PLK1
3	O04	control		UBC
3	O05	empty	empty	empty
3	O06	sample	M-004874-01	STK3
3	O07	empty	empty	empty
3	O08	sample	M-005382-00	STK31
3	O09	empty	empty	empty
3	O10	sample	M-005383-02	STK33
3	O11	empty	empty	empty
3	O12	sample	M-005384-00	STK35
3	O13	empty	empty	empty
3	O14	sample	M-005039-01	STK36
3	O15	empty	empty	empty
3	O16	sample	M-004674-00	STK38
3	O17	empty	empty	empty
3	O18	sample	M-003313-02	STK38L
3	O19	empty	empty	empty
3	O20	sample	M-004875-01	STK39
3	O21	empty	empty	empty

3	O22	sample	M-004157-01	STK4
3	O23	empty	empty	empty
3	O24	sample	M-003545-09	STK6
3	P01	empty	empty	empty
3	P02	empty	empty	empty
3	P03	control		PLK1
3	P04	control		UBC
3	P05	empty	empty	empty
3	P06	empty	empty	empty
3	P07	empty	empty	empty
3	P08	empty	empty	empty
3	P09	empty	empty	empty
3	P10	empty	empty	empty
3	P11	empty	empty	empty
3	P12	empty	empty	empty
3	P13	empty	empty	empty
3	P14	empty	empty	empty
3	P15	empty	empty	empty
3	P16	empty	empty	empty
3	P17	empty	empty	empty
3	P18	empty	empty	empty
3	P19	empty	empty	empty
3	P20	empty	empty	empty
3	P21	empty	empty	empty
3	P22	empty	empty	empty
3	P23	empty	empty	empty
3	P24	empty	empty	empty
